# Puromycin-sensitive aminopeptidase is involved in wild-type huntingtin clearance

**DOI:** 10.1186/1750-1326-7-S1-S5

**Published:** 2012-02-07

**Authors:** Guijie Ren, Zonghcai Ma, Maria Hui, Lili C Kudo, Koon-Sea Hui, Stanislav L Karsten

**Affiliations:** 1 Division of Neuroscience, Los Angeles Biomedical Research Institute at Harbor-UCLA Medical Center, Torrance, CA 90502, USA; 2 Department of Biochemistry and Molecular Biology, Medical College, Shandong University, Jinan, Shandong, 250012, China; 3 Nathan S. Kline Institute for Psychiatric Research, New York University School of Medicine, Orangeburg, NY 10962, USA; 4 NeuroInDx Inc., 1655 East 28th Street, Signal Hill, CA 90755, USA; 5 Department of Neurology, David Geffen School of Medicine at UCLA, Los Angeles, CA 90095, USA

## Background

Accumulation of neurotoxic mutated huntingtin protein (Htt) is an important contributing factor to the pathogenesis of Huntington’s disease (HD). More and more evidence has showed that wild-type Htt plays important roles in maintaining normal functions of neuronal system. The clearance of mutant Htt has been studied extensively; however, little is known about the mechanisms regulating accumulation and turnover of endogenous wild-type Htt. Puromycin-sensitive aminopeptidase (PSA) was recently identified as a major peptidase digesting TAU protein and protecting against TAU-induced neurodegeneration. PSA was also found as the peptidase responsible for digesting polyglutamine sequences released by proteasomes and removal of neurotoxic polyglutamine-expanded Htt exon-1, ataxin-3, mutant synuclein and superoxide dismutase 1 via autophagy system. These results suggest that PSA might represent a novel degradation mechanism targeting aggregate-prone neurotoxic protein substrates, including mutated Htt. However, the effects of PSA on endogenous wild-type Htt abundance remain unclear. Here, we investigated the effects of PSA on endogenous wild-type Htt clearance *in vivo* using a PSA overexpressing transgenic murine model and *in vitro* using human neuroblastoma cell line.

## Methods

We first determined whether PSA regulates Htt abundance *in vivo* using a human PSA (hPSA) transgenic mouse model, which was generated using bacterial artificial chromosome (BAC) mediated technology. For measuring the Htt expression in hPSA transgenic mice and control mice, the brain tissues were dissected and separated, and then were homogenized by sonication followed with centrifugation at 100,000 *g* at 4^o^C. Htt in supernatants were analyzed by Western blot using anti-Htt antibodies. Immunohistochemistry was also performed using perfused brains from both hPSA and control mice according to standard protocols. And we tested whether PSA overexpression is able to reduce the abundance of endogenous Htt in human neuroblastoma cells SH-SY5Y *in vitro*. SH-SY5Y cells were transfected with hPSA overexpression vectors pCMV6-XL-hPSA or blank vectors and then the endogenous Htt was analyzed with Western blot and immunocytochemistry. We also investigated the effect of inhibition of PSA expression using RNA interference (Stealth-RNAi) on endogenous Htt levels in SH-SY5Y cells. Cells were transfected with siRNA oligonucleotides specific for hPSA. Efficient hPSA silencing was confirmed with both real-time RT-PCR and Western blot analysis.

## Results

*In vivo* studies, Western blot analysis showed that Htt is significantly decreased in human PSA transgenic mice. Immunohistochemical experiment also showed Htt expression is decreased in all the transgenic brain regions tested. *In vitro* studies, Western blot analysis demonstrated decrease of endogenous Htt in response to PSA overexpression and accumulation of Htt in response to PSA slicing with Stealth-RNAi in human neuroblastoma cell line SH-SY5Y. And immunocytochemistry demonstrated notable decrease and rapid accumulation of Htt in response to PSA overexpression and PSA slicing respectively in SH-SY5Y cells.

## Conclusion

These combined *in vivo* and *in vitro* data implicate that PSA may be a key physiological regulator of clearance and turnover of wild-type Htt in neuronal system, and may play important roles in pathogenesis of HD through regulating clearance of wild-type or and mutated Htt as well.

